# Disordered Peptides Looking for Their Native Environment: Structural Basis of CB1 Endocannabinoid Receptor Binding to Pepcans

**DOI:** 10.3389/fmolb.2018.00100

**Published:** 2018-11-16

**Authors:** Alessandro Emendato, Remo Guerrini, Erika Marzola, Hans Wienk, Rolf Boelens, Serena Leone, Delia Picone

**Affiliations:** ^1^Department of Chemical Sciences, University of Naples Federico II, Naples, Italy; ^2^Department of Chemical and Pharmaceutical Sciences, University of Ferrara, Ferrara, Italy; ^3^Bijvoet Center for Biomolecular Research, Utrecht University, Utrecht, Netherlands

**Keywords:** pepcans, CB1 endocannabinoid receptor, endocannabinoid system, hemopressin, structure-activity relationships, intrinsically unfolded peptides

## Abstract

Endocannabinoid peptides, or “pepcans,” are endogenous ligands of the CB1 cannabinoid receptor. Depending on their length, they display diverse activity: For instance, the nona-peptide Pepcan-9, also known as hemopressin, is a powerful inhibitor of CB1, whereas the longer variant Pepcan-12, which extends by only three amino acid residues at the N-terminus, acts on both CB1 and CB2 as an allosteric modulator, although with diverse effects. Despite active research on their pharmacological applications, very little is known about structure-activity relationships of pepcans. Different structures have been proposed for the nona-peptide, which has also been reported to form fibrillar aggregates. This might have affected the outcome and reproducibility of bioactivity studies. In an attempt of elucidating the determinants of both biological activity and aggregation propensity of Pepcan-9 and Pepcan-12, we have performed their structure characterization in solvent systems characterized by different polarity and pH. We have found that, while disordered in aqueous environment, both peptides display helical structure in less polar environment, mimicking the proteic receptor milieu. In the case of Pepcan-9, this structure is fully consistent with the observed modulation of the CB1. For Pepcan-12, whose allosteric binding site is still unknown, the presented structure is compatible with the binding at one of the previously proposed allosteric sites on CB1. These findings open the way to structure-driven design of selective peptide modulators of CB1.

## Introduction

The name “pepcans” (endocannabinoid peptides) refers to a family of hemoglobin-derived peptides that act as endogenous ligands of the cannabinoid (CB) receptor CB1 (Bauer et al., 2012; Macedonio et al., 2016). This G-protein coupled receptor (GPCR) is widely expressed in the nervous system and, together with the CB2 receptor, mostly expressed by immune cells, is the primary target of endocannabinoids, such as anandamide and 2-arachidonoylglylcerol (2-AG), and of phytocannabinoids, among which Δ^9^-tetrahydrocannabinol (THC), the main active component of marijuana (Mechoulam and Parker, 2013; Macedonio et al., 2016). The ensemble of endocannabinoids and their receptors constitutes the Endocannabinoid System (ECS), which is involved in a wide range of physiological processes and manifestations, including cognitive function, pain, anxiety and appetite regulation (Pagotto et al., 2006; Watkins and Kim, 2015; Reddy and Golub, 2016; Leone et al., 2017). A great wealth of evidence has also linked the ECS to neuroprotection and symptom reduction in models of many neurological diseases, including multiple sclerosis, Alzheimer's and Parkinson's (van der Stelt and Di Marzo, 2005; van der Stelt et al., 2006; Price et al., 2009; Chiurchiù et al., 2018). Thus, modulation of CB1 and CB2 response by means of selective compounds poses a stimulating challenge for drug design, also in light of the observed psychoactive effects of the first generation of synthetic cannabinoid drugs, which have led to their withdrawal from the market (Cridge and Rosengren, 2013).

Typically, both endogenous and synthetic CB ligands are small lipophilic molecules, with little common structural features. Pepcans were discovered only 15 years ago (Rioli et al., 2003) and their CB1 modulating activity was detected even more recently (Heimann et al., 2007). Being the first reported peptide modulators of the cannabinoid receptor, they have sparked much interest, opening the possibility to develop peptide-based scaffolds for pharmaceutical applications (Bomar and Galande, 2013; Macedonio et al., 2016). Pepcan-9, also known as hemopressin, was the first member of the family to be identified in rat brain homogenates. It is a nona-peptide with amino acid sequence PVNFKFLSH, corresponding to amino-acids 96–104 of the α-chain of hemoglobin (Heimann et al., 2007; Gomes et al., 2010). Pepcan-9 was found to be a selective inverse agonist of CB1, with an activity and a binding efficiency comparable to that of the synthetic CB1 inhibitor rimonabant (Heimann et al., 2007). The human homolog differs from the murine peptide only by a Phe/Leu mutation at position 6, and has similar activity (Bauer et al., 2012). Pepcan-9 administration elicits a marked antinociceptive response (Heimann et al., 2007; Toniolo et al., 2014) and produces hypotensive effects and reduction of the food intake in animal models (Blais et al., 2005; Dodd et al., 2010). Besides Pepcan-9, other N-terminal extended hemopressin peptides have been isolated, referred to as Pepcan-11 to Pepcan-23, according to their length (Bauer et al., 2012). Indeed, experimental evidences suggest that, despite the observed bioactivity, Pepcan-9 does not exist as such *in vivo*, being rather a purification artifact of longer homologs, generated upon cleavage of an extremely labile Asp-Pro peptide bond upon hot acid extraction (Marcus, 1985; Gomes et al., 2010; Bauer et al., 2012; Bomar and Galande, 2013). Independently from its existence *in vivo*, the marked bioactivity, coupled to the possibility of obtaining it with synthetic approaches, makes it interesting in the perspective of potential pharmacological uses. Among the longer peptide variants, the most abundant and active is Pepcan-12 (RVD-hemopressin), which is expressed and released in the central nervous system by noradrenergic neurons (Hofer et al., 2015). Initial studies suggested that this peptide exhibited agonist activity toward CB1 (Gomes et al., 2009), but later evidences supported instead the view of Pepcan-12 as a negative allosteric modulator (NAM) of CB1 (Bauer et al., 2012; Straiker et al., 2015). A recent study by Petrucci et al. has demonstrated that the peptide is also constitutively secreted in adrenals and liver upon tissue damage and acts as a positive allosteric modulator (PAM) of CB2 as well (Petrucci et al., 2017). Pepcan-12 is one of the few known endogenous allosteric modulators of the CB receptors (Morales et al., 2016). As allosteric modulation might be a mean to fine tune, or even overcome, the adverse psychoactive effects of some cannabinoid drugs, the understanding of the molecular mechanisms of the phenomenon constitute a hot topic in pharmaceutical research (Khurana et al., 2017). The allosteric binding site for Pepcan-12 is still unknown, whereas, since the introduction of chemical modifications at the N-terminus greatly reduced its affinity toward CB1, this latter portion is likely responsible for receptor binding (Bauer et al., 2012). Exogenous administration of Pepcan-12 induces anorexigenic and antinociceptive effects in rat models, with little or no side effects (Han et al., 2014; Ferrante et al., 2017), and has been able to restore impaired memory functions in Aβ_1−42_ treated mice, highlighting its pharmacological versatility also in the treatment of Alzheimer Disease-induced memory deficits (Zhang et al., 2016). Given the proven involvement of the ECS in several neurodegenerative diseases, the full pharmacological potential of pepcans may yet to be fully revealed.

Despite all these promising findings, work with pepcans in drug development has been hampered by the low reproducibility of the pharmacological assays (Gomes et al., 2009), which has been partially attributed to a certain propensity to self-aggregation: for instance, under physiological conditions, Pepcan-9 forms amyloid-like nanostructured fibrils, which may precipitate from the solution, leading to inconsistent activity reports (Bomar et al., 2012). Fibrillation has never been observed for Pepcan-12 in similar conditions, despite the fact that these pepcans differ only by the RVD extension at the amine terminus. Additionally, little is known about the conformational properties of pepcans in solution. Previous NMR studies on Pepcan-9 and its bioactive derivative, the C-terminally truncated hexa-peptide (PVNFKF), employed DPC/SDS micelles to reproduce the lipophilic receptor environment (Scrima et al., 2010). These conditions induced the formation of regular β-turn structures in the peptides, and docking studies on a homology model of CB1 suggested that such conformations could explain the binding to the same pocket as rimonabant (Scrima et al., 2010). Very recently, the structure of the CB1 receptor has been resolved by X-ray crystallography, in complex with both agonist and antagonist compounds (Hua et al., 2016, 2017; Shao et al., 2016). These structures have revealed that the receptor is endowed with intrinsic plasticity and undergoes sensible conformational changes correlating with its activation. Several hydrophobic interactions with orthosteric ligands were detected, differing from those proposed on the basis of the homology model. In the present study, we describe the thorough structural characterization of Pepcan-9 and Pepcan-12 with a dual intent: to understand and overcome the aggregation propensity of the peptides, we explored their conformational behavior both in water and the presence of an apolar fluorinated alcohol, hexafluoroisopropanol (HFIP), as a mean to mimic the low polarity of the receptor milieu. Both acidic and neutral pH were employed, to understand whether the protonation state of the peptide could drive the aggregation process. Finally, we used docking studies to understand the possible binding modes leading to differential CB1 activity modulation.

## Materials and methods

### Materials

Pepcan-9 and Pepcan-12 were synthesized by solid phase peptide synthesis as previously reported (Remelli et al., 2016). HFIP, both protonated and deuterated, and phosphate buffers were purchased at the highest available purity from Sigma-Aldrich (St. Louis, Missouri, USA) and used without further purification.

### CD spectroscopy

Stock solutions of Pepcan-9 and Pepcan-12 were prepared at 2 mM concentration in 10 mM HCl. Peptides solutions were diluted in 20 mM sodium phosphate buffer (NaP) at pH 3.0 or 7.4 to the final concentration of 20 μM. CD spectra were recorded on a Jasco J-715 spectropolarimeter (Jasco international Co. Ltd, Tokyo, Japan) using a 0.1 cm path length quartz cuvette at 20°C in continuous scanning mode (20 nm/min, with a 4.0 s response and a 1.0 nm band width). Three acquisitions were averaged and the solvent contribution was subtracted for each spectrum. The results are expressed as molar ellipticity [θ] (deg cm^2^/mol). Deconvolution analyses were performed with the BestSel software (Micsonai et al., 2015).

### NMR spectroscopy and structure calculation

Stock peptide solutions were diluted to a final concentration of 500 μM in 20 mM NaP or in 50% v/v HFIP/NaP, at pH 3.0 or 7.4, containing 5% D_2_O. 1D and 2D NMR experiments were carried out at 298 K on a 900 MHz Bruker AVANCE III instrument equipped with a cryogenic probe. TOCSY experiments used a mixing time of 80 ms, NOESY spectra were acquired with 200 and 300 ms mixing time, and ROESY spectra used a 100 ms spinlock. 2D spectra of 2048 × 512 complex points (12 × 12 ppm) were recorded with 32 scans. DQF-COSY was recorded with 48 scans and 2048 × 256 complex points; ^1^H,^13^C-HSQC was recorded using 256 scans, 1024 × 256 complex points and a ^13^C-frequency range from 0 to 80 ppm. All spectra were processed using zero-filling, linear prediction for the indirect dimension of the DQF-COSY, TOCSY and ^1^H,^13^C-HSQC spectra, and squared cosine apodization in both dimensions. All chemical shifts were referenced with respect to the on-resonance water signal. Spectra were processed using NMRPipe (Delaglio et al., 1995). Data assignment and analysis were performed using the CCPN suite (Vranken et al., 2005). NOE contacts were converted to distance restraints with CCPN own routine and dihedral restraints were inferred using CCPN implementation of DANGLE (Cheung et al., 2010). The peptides structure models were obtained using ARIA (Rieping et al., 2007). To calculate structures for Pepcan-9 at pH 3.0, 76 unambiguous distance restraints and 8 dihedral restraints were used (60 and 10, respectively, for Pepcan-9 at pH 7.4). For Pepcan-12 at pH 3.0, 70 unambiguous distance restraints and 16 dihedral restraints were used (59, and 16, respectively for Pepcan-12 at pH 7.4). Seven simulated annealing cycles were performed with 200 structures/step. At the end of each step the lowest energy structures were used to automatically improve the peak assignment for the next cycle. On the eighth iteration, 100 structures were calculated. Of these, the 10 lowest energy structures were selected and water-refined.

### Docking calculations

The lowest energy structure from the NMR ensembles obtained for both peptides in NaP/HFIP at pH 7.4 was used for docking studies. PDB structure 5U09 (Shao et al., 2016) was used as a model for the CB1 receptor. Docking experiments were performed with Autodock 4.2 (Morris et al., 2009) with the Lamarckian Genetic Algorithm (LGA), using Kollman charges for both the receptor and the peptide. The receptor and the peptide backbone were kept rigid, the peptide side chains were allowed to rotate. 100 runs were performed with an initial population of 500 structures, a maximum number of 2.5 × 10^6^ energy evaluations, and of 2.7 × 10^5^ generations. Refinement of the lowest energy structure of the Pepcan/CB1 complexes was achieved by *in vacuo* energy minimization with the steepest descent in GROMACS 5.1.4 (Pronk et al., 2013), and the minimized complexes were re-docked with 100 rounds of local search. Protein-protein docking to define possible allosteric binding sites for Pepcan-12 was performed with the web implementation of FRODOCK (Ramírez-Aportela et al., 2016)

## Results and discussion

### Conformational analysis by CD spectroscopy

The conformation of both peptides in aqueous environment at acidic and neutral pH, i.e., in 20 mM sodium phosphate buffer (NaP) at pH 3.0 and 7.4, was analyzed by Circular Dichroism (CD) spectroscopy. The spectra (Figure S1) suggest that their structure is mostly disordered, as expected for small size linear peptides in aqueous solution (Pastore and Temussi, 2013). Deconvolution of the spectra indicated a α-helix content of 0 and 6% for Pepcan-9, and 30 and 15% for Pepcan-12 at neutral and acidic pH, respectively, indicating that the longer peptide is always more structured than the shorter one, in particular at neutral pH.

To understand if such conformational preferences are maintained also in a less polar environment, as would be the case at the receptor binding site, the conformation of the peptides was also analyzed in solvent mixtures containing 0 to 100% hexafluoro-isopropanol (HFIP), at both pH values. It is well-known that the low polarity of the NaP/HFIP solutions promotes *intra*-peptide interactions over peptide-solvent interactions, and HFIP has been widely used to highlight secondary structure propensity in hydrophobic environments (Crescenzi et al., 2002; Tomaselli et al., 2006; Bernardi et al., 2010; Aschi et al., 2017).

**Figure 1:**
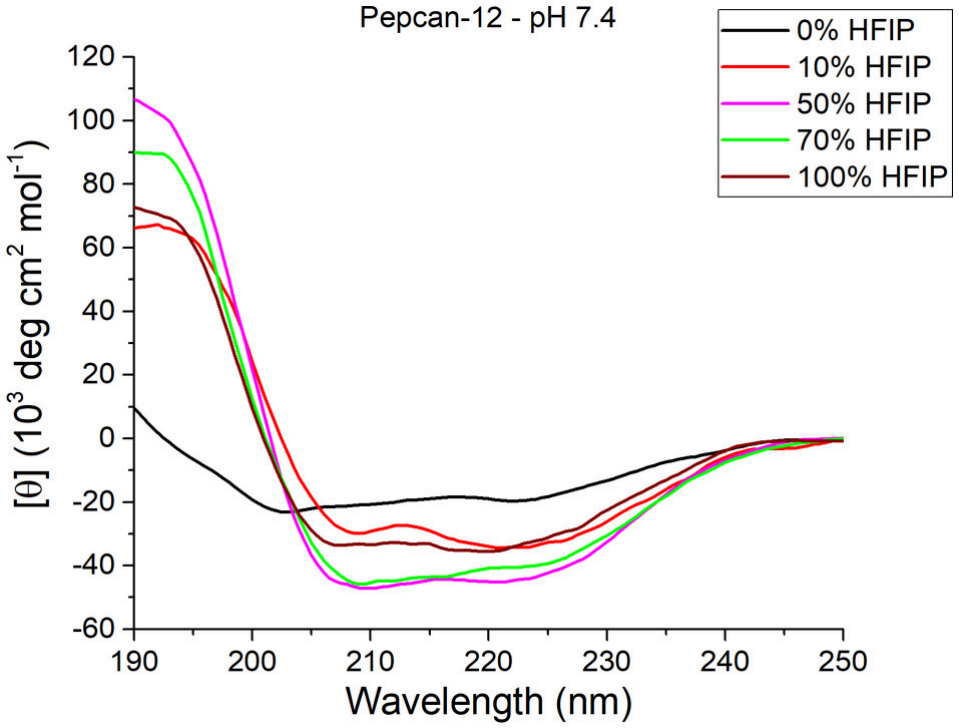
CD spectra of Pepcan-12 in different HFIP/ NaP mixtures, pH 7.4. The appearance of a signal diagnostic of helical secondary structure is visible in the presence of as little as 10% HFIP.

As expected, HFIP significantly increases the helical content in both peptides. The most significant effects were detected for Pepcan-12 at pH 7.4 (Figure 1), for which even the presence of as little as 10% of HFIP leads to substantial spectral changes, pointing to an increase of helical structure (Figure 1, red vs. black lines). The spectra recorded in the other conditions are reported in the supplementary material (Figure S2).

**Figure 2:**
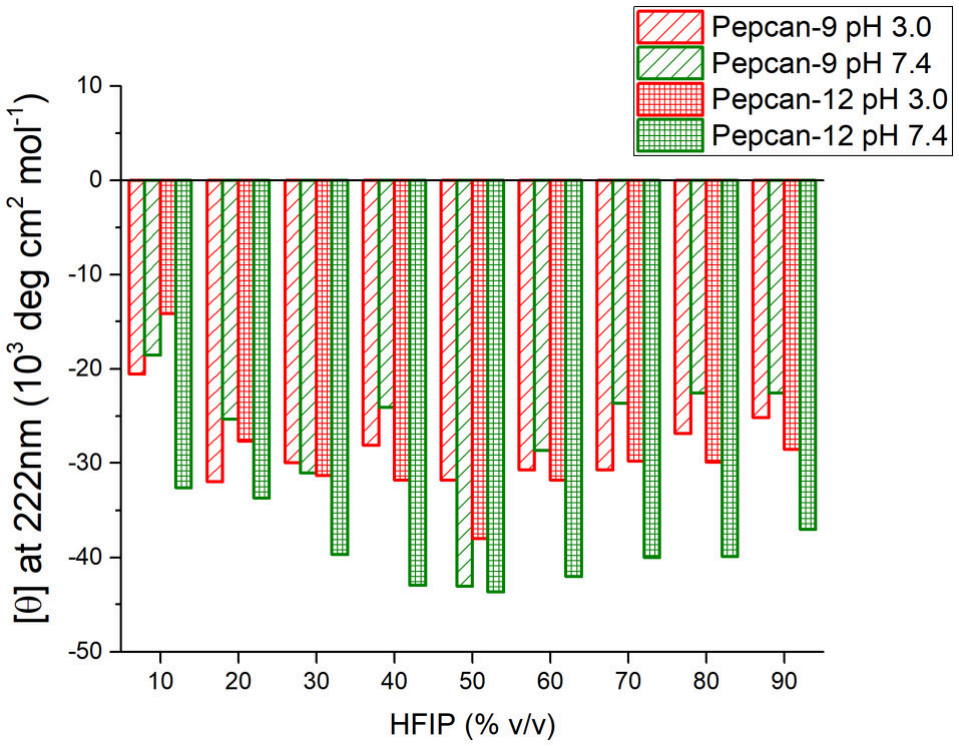
Molar ellipticity per residue measured at 222 nm (canonical minimum of α-helix conformation) as function of HFIP content in the mixture, for all conditions studied.

Similar but smaller effects were observed for Pepcan-9, where, upon dissolution in 10% HFIP in NaP, the helical content raised to 30 %. Monitoring the ellipticity at 222 nm as a marker of the peptide helicity, the highest helical content was observed between 40 and 70% HFIP, and decreased at higher HFIP concentrations (Figure 2). Both peptides at both pHs exhibited similar trends (Figure 2).

### NMR analysis

The two peptides were subjected to NMR characterization in either aqueous or low polarirty environment, and at both pH values. Based on the results of the CD analysis, we selected as a suitable low polarity condition the 50/50 NaP/HFIP system, both peptides appear significantly structured.

A full set of two-dimensional NMR experiments (TOCSY, NOESY, ROESY, DQF-COSY, and ^1^H,^13^C-HSQC) were employed for proton and carbon resonances assignment. The ^1^H and ^13^C resonances assigned are reported in Tables S1–S8 of the supplementary material. For structural comparison, the residue numbering of Pepcan-12 was used in all cases.

In aqueous medium, at both pH values, the spectra exhibit the typical behavior of intrinsically disordered peptides (Temussi et al., 1989; Balboni et al., 1990; Pastore and Temussi, 2013), i.e., poor signal dispersion and no NOE contacts other than sequential ones (data not shown). Upon dissolution in 50/50 NaP/HFIP, good quality NMR spectra were obtained. Diagnostic regions of the NOESY and TOCSY spectra for both peptides at pH 3.0 are reported in Figure S3 of the supplementary material, together with NOE medium-range contact overviews bar diagrams obtained under all conditions (Figure S4).

At pH 3.0, diagnostic NN i,i+1 and αN i,i+3 contacts suggested the presence of helical structure at the C-terminus of both peptides, with a longer helical segment in Pepcan-12. Furthermore, the Pro4 was found in the *trans* conformation.

**Figure 3:**
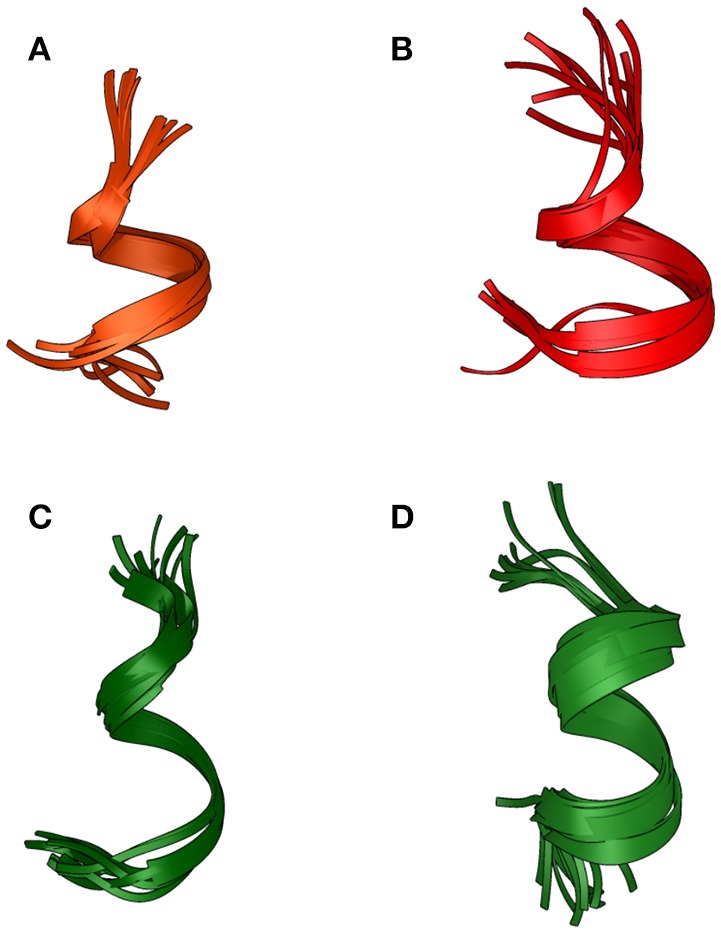
Backbone overlay of the 10 lowest energy NMR-conformations of Pepcan-9 **(A)** and Pepcan-12 **(B)** in 50/50 v/v HFIP/NaP, pH 3.0, and Pepcan-9 **(C)** and Pepcan-12 **(D)** in 50/50 v/v HFIP/NaP, pH 7.4.

Based on NOE derived distance restrains and chemical shifts data, the structures of the two peptides were generated (Figure 3): a short helical region is present at the C-terminus of Pepcan-9, between residues 6 and 11 (Figure 3A), whereas Pepcan-12 exhibits a helix spanning from Val5 to Ser11 (Figure 3B).

At pH 7.4, fewer and weaker NOE contacts were observed for both peptides. For Pepcan-9, diagnostic signals for His12 were no longer observed. Accordingly, the calculated structures suggest the presence of a helical region in the stretch Asn6-Leu10, while the C-terminal Ser and His are disordered (Figure 3C). In the case of Pepcan-12, the calculated structure present a helical region spanning from Asp3 to Ser11 (Figure 3D).

Our NMR data indicate that pH has opposite effects on the two peptides: for Pepcan-9, a helical structure is favored at acidic pH, whereas for Pepcan-12 a helical structure is favored at neutral pH. This finding can be explained on the basis of the protonation states of His12 (present in both peptides) and Asp3 (present only in Pepcan-12). At pH 3.0, the side-chain of His12 is protonated, stabilizing the helical structure at the C-terminus (C-capping). This can not happen at pH 7.4, where the His12 sidechain is likely uncharged. In the case of Pepcan-12 this effect is paralleled by the stabilizing influence of the Asp negative charge at the N-terminal site (N-capping), which induces a shift of the helix region. Such further helix stabilization is not possible for Pepcan-9.

HFIP exerts its described helix-stabilizing effect when present up to 60%, but further decreasing the medium polarity has detrimental effects on the peptide structure. In fact, the decrease of the dielectric constant can have a dramatic influence on the pK_a_ of amino acid side-chains (Spadaccini et al., 2016), due to the stabilization of uncharged states, which, in this case, would reduce the positive influence of both helix capping effects.

**Figure 4:**
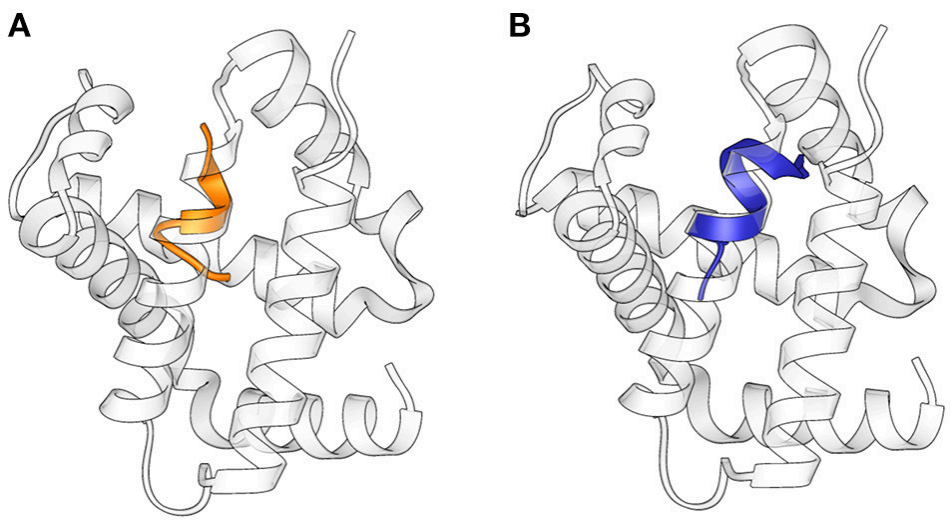
Superimposition of the structures of (**A)** Pepcan-9 (orange) and **(B)** Pepcan-12 (blue) on the α-chain of hemoglobin (PDB code 2DN1).

The structures obtained in our studies closely resemble the native conformation of the peptides within the α-chain of hemoglobin (Figure 4) (Park et al., 2006): Cα RMSD between corresponding residues in the folded stretch of the peptides and within the protein structure were 0.647 and 0.496 Å for Pepcan-9 and Pepcan-12, respectively, indicating that the 50/50 NaP/HFIP solvent system can correctly mimic the environment of a protein interior.

On the other hand, our structures differ substantially from earlier literature reports employing other systems: previous studies had elucidated the structure of the rat homolog of Pepcan-9 in DPC/SDS micelles (Scrima et al., 2010), identifying a regular type I β-turn at the C-terminus of the peptide and a substantially disordered N-terminal end. This peptide β-sheet propensity had also been detected in the presence of 25% trifluoroethanol (TFE), although no structure could be derived in these conditions (Bomar et al., 2012).

### Docking studies

**Figure 5:**
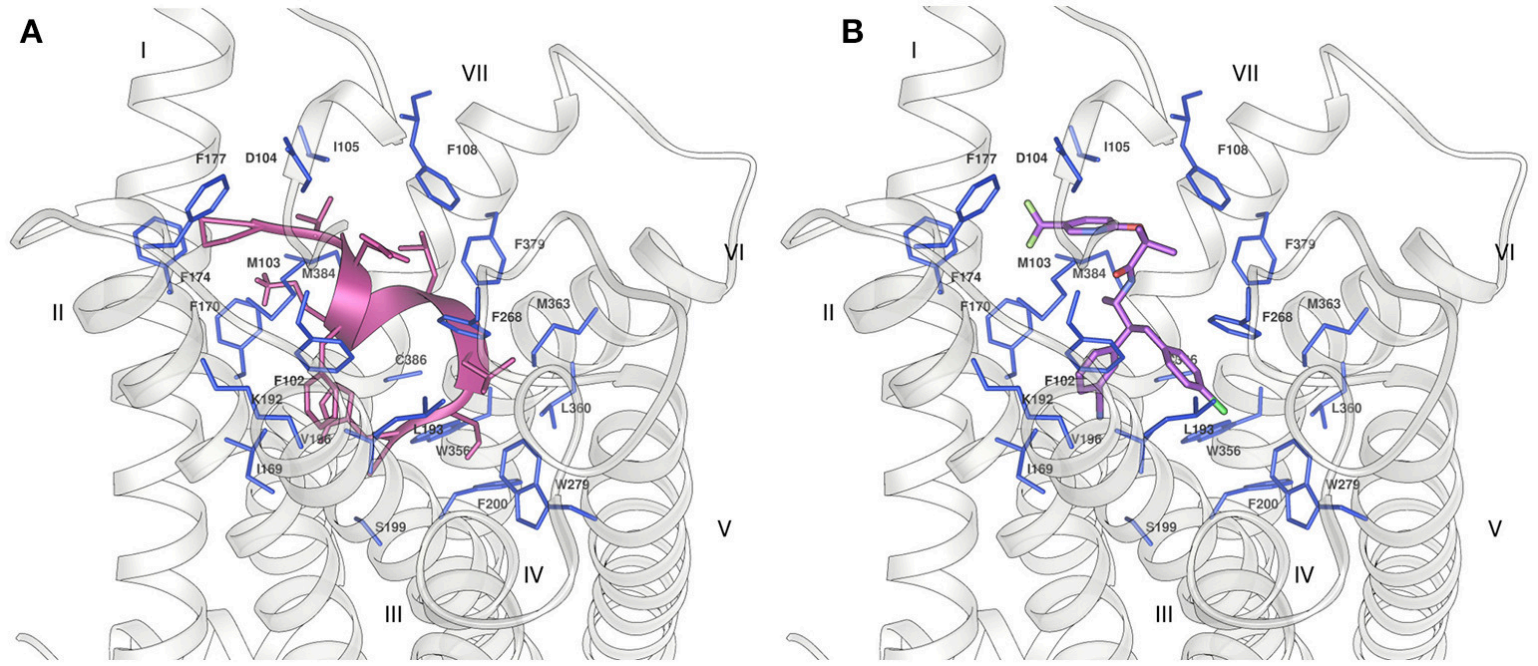
Binding of **(A)** Pepcan-9 (pink) and **(B)** taranabant (purple) to the CB1 receptor (grey ribbon) (PDB code 5U09). The residues of the receptor in close proximity to the ligand are displayed as blue sticks.

In order to understand whether our NMR structures could correlate to the different bioactivity of Pepcan-9 and 12, we performed peptide docking on the crystal structure of the CB1 receptor. Pepcan-9 has an antagonist/ inverse agonist effect on CB1, with a binding affinity and inhibition constant very similar to those of the synthetic inhibitor rimonabant (Heimann et al., 2007). The structure of CB1 in complex with the inhibitor taranabant, a close relative of rimonabant, has recently been resolved (PDB code 5U09) (Shao et al., 2016), and docking studies in that framework indicated that taranabant and rimonabant have similar binding modes, involving a binding pocket located toward CB1 transmembrane helices TM1 and TM7 (Shao et al., 2016). We used our lowest energy structure of Pepcan-9 in the low polarity solvent system and at physiological pH to perform docking studies on CB1. To preserve the structure based on NMR restraints, during the docking the peptide backbone was kept rigid, whereas all side chains were allowed to rotate freely. Despite the low resolution of this approach, which did not take into account the flexibility of both binding partners, the docking results show that the NMR conformation of Pepcan-9 matches the taranabant binding pocket, with a calculated binding energy of −10.81 kcal/mol (Figure 5). All the residues essential for taranabant binding are in proximity of the side chains of Pepcan-9. In particular, Pro4 is in the vicinity of Met103, Phe 174, and Phe177; Phe7 interacts with Phe102, Met103, Ile169, and Val196 of CB1; Leu9 of Pepcan-9 is in contact with the side chains of Phe108 and Phe379, Leu10 with Trp279, Leu359, and Met363. Additionally, a favorable cation-π interaction can form between the side chain of Lys8 and Phe170. This extensive interaction pattern would explain the selectivity that is exhibited by Pepcan-9 toward CB1 and its high inhibition constant, meanwhile supporting the fact that the NMR structure in the 50/50 NaP/HFIP solvent system could closely resemble that of the peptide in its bioactive form.

**Figure 6:**
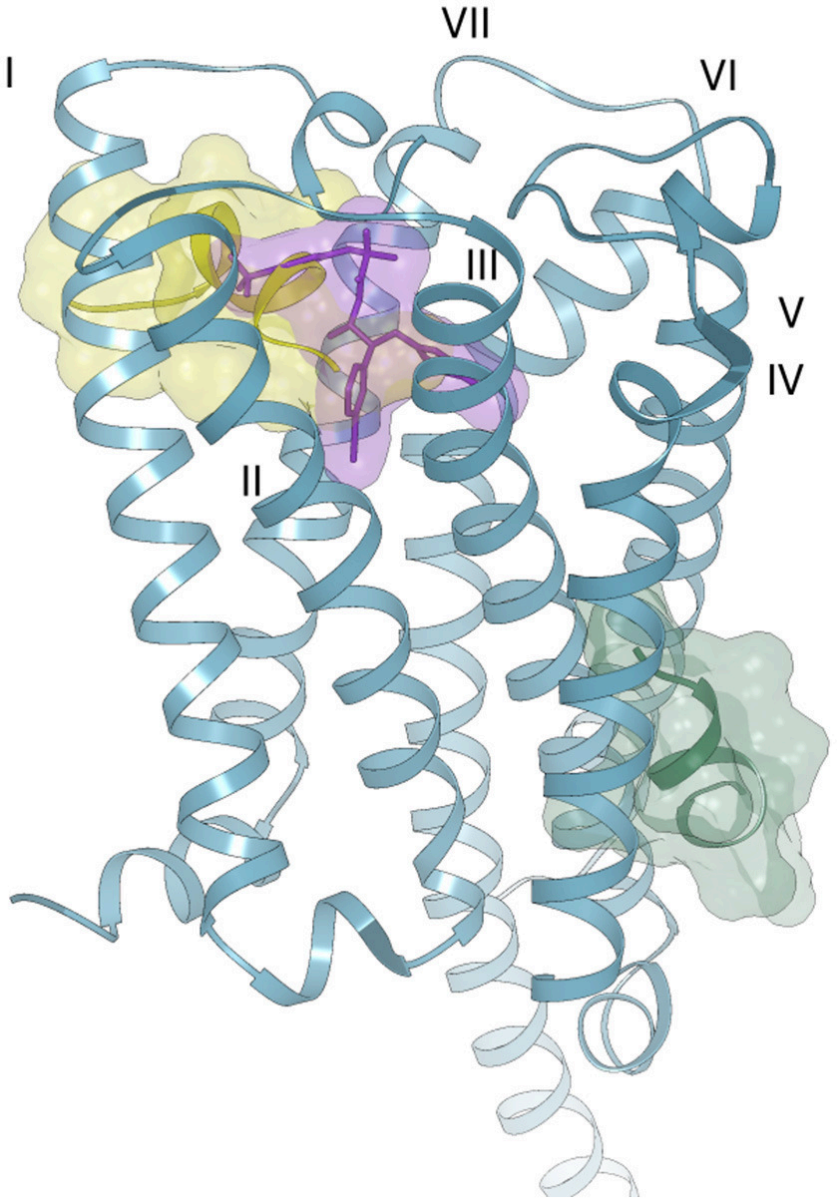
Possible binding modes of Pepcan-12 (yellow and green surfaces) to the CB1 receptor (blue ribbon) (PDB code 5U09). The orthosteric binding site is highlighted by the presence of a taranabant molecule (purple stick and surface).

In the case of Pepcan-12, the same approach could not be applied, due to the absence of experimental data on the peptide binding site. Indeed, despite the sequence difference of only three aminoacids, attempts of docking in the orthosteric binding pocket produced only complexes with positive binding energies, due to the occurrence of severe clashes between Pepcan-12 and CB1, as indicated in Figure S5. Several allosteric modulators have been identified for CB1, but the location of their binding site (or sites) on the receptor has yet to be clarified (Khurana et al., 2017). It should also be noted that the binding of allosteric modulators is often dependent on the nature of the orthosteric ligand, further complicating structural predictions. To date, the best characterized allosteric modulator of CB1 is the synthetic compound ORG27569. Yet, different studies have suggested discordant binding pockets: based on fluorescence labeling studies, a site proximal to the extracellular membrane and comprising the disulfide bond Cys98-Cys107 has been proposed in combination with the orthosteric ligand CP55,940 (Fay and Farrens, 2013). Mutagenesis and molecular modeling studies have also pointed toward a binding site partially overlapping with that of rimonabant and close to the N-terminus, involving the interaction with residues K192, F200, W279, and W356 of the TM helices, as well as with F268 of the EC2 extracellular domain (Shore et al., 2014). This partial overlap of the orthosteric and allosteric binding sites could explain receptor modulation through alterations in the dissociation kinetics of the primary ligand, with variable outcomes depending on the orthosteric ligand. A different site, in the intracellular portion of TM1, TM2 and TM4 has instead been proposed on the basis of computational methods, cross-linking and mass spectrometry analysis (Stornaiuolo et al., 2015). This site was selected out of 5 potential binding sites unique to CB1, in turn chosen among 9 potential binding sites common to both CB1 and CB2, on a homology model of CB1. Given the peptide nature of Pepcan-12, it is plausible that it might employ yet another binding pocket, compared to synthetic allosteric modulators. Both molecular dynamics (Shore et al., 2014; Stornaiuolo et al., 2015) and the comparison of the crystal structures of CB1 bound to diverse agonists/ antagonists orthosteric ligands (Hua et al., 2016, 2017; Shao et al., 2016) have suggested a high intrinsicplasticity of CB1: the crystal structures reveal that the orthosteric binding site is ~50% smaller in the case of agonist binding than in antagonist binding, due to significant movements of TM1 and TM2 (Hua et al., 2017). This plasticity could be further influenced by the concomitant binding of allosteric modulators, in ways that are yet to be completely elucidated. Additionally, peptides are extremely flexible molecules, and the conformational space of the ligand should also be explored when performing blind docking experiments. For all these reasons, the definition of the binding interactions between Pepcan-12 and CB1 by computational methods, in the absence of strong experimental data, poses a considerable challenge. At any rate, to get a hint on whether the above described NMR structure of Pepcan-12 possessed any biological significance, we used a simplified approach, trying to predict its possible binding site by rigid body docking and shape complementary on the protein surface. Blind docking experiments on the crystal structure of CB1 revealed only two likely binding sites: the first (Figure 6, yellow surface), is located toward the extra-cellular portion, partially overlapped with the taranabant binding site, whereas the second (Figure 6, green surface) encompassing a lipid exposed region of TM3, TM4, and TM5. The first site would be in agreement with previous reports (Fay and Farrens, 2013, 2015; Shore et al., 2014). Moreover, this solution seems the most plausible, given the peptidic nature of the ligand, which would necessitate active ligand transport across the membrane. Although it is possible that the actual complex between Pepcan-12 and CB1 will involve yet a different site, and/or conformational state, and/or some extent of induced fit of both partners, our NMR structure seems therefore a good starting point for further structural and computational investigations on this complex activation mechanism.

## Discussion

In an attempt to define the structural determinants of their bioactivity and aggregation propensity, we have analyzed the conformational properties of the two most representative endocannabinoid peptides, Pepcan-9 and Pepcan-12. Pepcan-12 is an allosteric modulator of both CB1 and CB2 receptors, and is produced and released in a variety of tissues, also in response to cellular stress. The existence of Pepcan-9 *in vivo* has instead been questioned: experimental data indicate that it could be a purification artifact from the cleavage of longer peptides. Nonetheless, many studies have analyzed the effects of exogenous administration of Pepcan-9, which can also be produced by synthesis, revealing its interesting pharmacological profile, for instance in pain and appetite modulation. While in aqueous environment both peptides are unstructured, the presence of a low polarity environment increases the content of α-helical structure and emphasizes conformational differences between the peptides. In particular, in a 50/50 HFIP/NaP/buffer system, the structure of both peptides resembles the native conformation at the N-terminal region of the helix G of hemoglobin. This validates the idea that such solvent system has potential to mimic the protein interior. At acidic pH, both peptides present a helical region at the C-terminus. At neutral pH, the helical region is longer, but slightly shifted toward the N-terminus in Pepcan-12, whereas it is reduced to a single turn in Pepcan-9. We have proposed that these latter structures may resemble the peptide bioactive conformations *in vivo*. Indeed, by means of docking studies, we have demonstrated that, in the described conformation, Pepcan-9 can efficiently bind to CB1, suggesting a bioactivity similar to that of rimonabant. In the case of Pepcan-12, the results could not be as detailed as for Pepcan-9, due to the lack of knowledge on its precise binding pocket on CB1. Rigid body docking of the Pepcan-12 NMR structure on the receptor surface led to the detection of two possible binding sites. The first and most likely is located toward the extracellular portion of CB1 and partially overlaps with the orthosteric binding site, in accordance with the results of previous studies on another CB1 allosteric modulator, ORG27569 (Fay and Farrens, 2013; Shore et al., 2014). The second binding pocket is instead located in a lipid exposed portion of TM3, TM4 and TM5, but it seems unlikely a relevant binding location as translocation mechanisms would be needed for the peptide to reach it. This docking approach does not allow more detailed predictions of molecular interactions and additional experimental data are still needed to clarify the precise binding mode of Pepcan-12 to CB1. Nevertheless, as one of the putative allosteric binding sites of CB1 was indeed detected, it seems that our results can still provide the structural basis to design peptide ligands for CB1. It is also interesting to highlight that, during our experiments, including a period of several days needed for NMR experiment acquisition, no aggregation nor precipitation was observed for either peptide in any condition explored. This is in apparent contrast with previous literature reports on formation of fibrils by Pepcan-9 (Bomar et al., 2012). In those reports, the conformational properties of Pepcan-9 were studied upon the addition of 25% TFE, a condition known to promote secondary structure, which led to the observation of β-like structure. Similarly, a β-turn characterized the structure of Pepcan-9 in DPC/SDS micelles. Possibly, the helical and β-strand forms of Pepcan-9 are coexistent, and the preference toward either conformation could direct the aggregation behavior of Pepcan-9, interfering with its CB1 activity. We hypothesize that peptide modifications aimed to stabilize the described helical conformation and avoid β-strand formation and aggregation might be a first step in the development of selective peptide-based CB1 ligands.

## Author contributions

AE performed structural studies, SL performed computational studies. RG and EM provided the peptides. HW acquired the NMR spectra. DP helped with NMR analysis and coordinated the work. AE, SL, and DP wrote the paper. All authors discussed the results and approved the manuscript.

### Conflict of interest statement

The authors declare that the research was conducted in the absence of any commercial or financial relationships that could be construed as a potential conflict of interest.
