# Supplementary material for: Disordered Peptides Looking for Their Native Environment: Structural Basis of CB1 Endocannabinoid Receptor Binding to Pepcans
Source: Front Mol Biosci. 2018 Nov 16;5:100. doi: 10.3389/fmolb.2018.00100 (PMC6250848; doi:10.3389/fmolb.2018.00100)
Supplement: Supplementary file 1 [file Data_Sheet_1.docx]

Supplementary Material

Disordered peptides looking for their native environment: Structural basis of CB1 endocannabinoid receptor inhibition by pepcans

Alessandro Emendato^1^, Remo Guerrini^2^, Erika Marzola^2^, Hans Wienk^3^, Rolf Boelens^3^, Serena Leone^1*^ and Delia Picone^1*^

*** Correspondence:**

Dr. Serena Leone: [serena.leone@unina.it](mailto:serena.leone@unina.it)

Prof. Delia Picone: [delia.picone@unina.it](mailto:delia.picone@unina.it)

**Supplementary Table S1: ^1^H and ^13^C chemical shifts of Pepcan-9 in NaP pH 3.0**

| Residue | NH | αH | βH | δH | εH | γH | zH | αC | βC | δC | εC | γC |
| --- | --- | --- | --- | --- | --- | --- | --- | --- | --- | --- | --- | --- |
| 4-Pro |  | 4.34 | 2.37, 1.93 | 3.31 |  | 1.97, 1.91 |  | 62.21 | 32.60 | 49.38 |  | 26.42 |
| 5-Val | 8.48 | 3.96 | 1.85 |  | 0.80, 0.71 |  |  | 62.61 | 32.73 |  |  | 20.44, 20.97 |
| 6-Asn | 8.41 | 4.61 | 2.71, 2.63 | 7.52 |  |  |  |  | 38.76 |  |  |  |
| 7-Phe | 8.15 | 4.44 | 2.94, 3.04 | 7.16 | 7.27 |  | 7.23 | 58.26 | 39.53 |  |  |  |
| 8-Lys | 8.06 | 4.13 | 1.67, 1.62 | 1.57 | 2.89 | 1.26 | 7.44 | 56.44 | 32.90 | 29.04 | 42.04 | 24.73 |
| 9-Leu | 7.91 | 4.22 | 1.52 | 0.86, 0.80 |  | 1.51 |  | 55.08 | 42.24 | 24.79, 23.52 |  | 26.94 |
| 10-Leu | 8.04 | 4.30 | 1.49, 1.57 | 0.83, 0.77 |  | 1.54 |  | 54.94 | 42.32 | 24.96, 23.30 |  | 26.84 |
| 11-Ser | 8.07 | 4.32 | 3.75 |  |  |  |  | 58.19 | 63.87 |  |  |  |
| 12-His | 8.15 | 4.52 | 3.21, 3.07 | 8.49 | 7.19 |  |  | 55.89 | 29.46 |  |  |  |

**Supplementary Table S2: ^1^H and ^13^C chemical shifts of Pepcan-9 in NaP pH 7.4**

| Residue | NH | αH | βH | δH | εH | γH | zH | αC | βC | δC | εC | γC |
| --- | --- | --- | --- | --- | --- | --- | --- | --- | --- | --- | --- | --- |
| 4-Pro |  | 4.31 | 2.35, 1.91 | 3.28 |  | 1.89, 1.95 |  | 62.19 | 32.64 | 49.37 |  | 26.49 |
| 5-Val |  | 3.96 | 1.86 |  | 0.80, 0.71 |  |  | 62.55 | 32.71 |  |  | 20.43, 20.98 |
| 6-Asn |  |  | 2.71, 2.62 |  |  |  |  |  | 38.82 |  |  |  |
| 7-Phe |  | 4.46 | 2.92, 3.03 |  |  |  |  | 58.01 | 39.55 |  |  |  |
| 8-Lys |  | 4.14 | 1.67, 1.60 | 1.56 | 2.88 | 1.24 |  | 56.29 | 33.0 | 29.05 | 42.05 | 24.70 |
| 9-Leu | 7.96 | 4.21 | 1.51 | 0.85, 0.79 |  | 1.49 |  | 55.13 | 42.27 | 24.80, 23.55 |  | 26.99 |
| 10-Leu | 8.06 | 4.30 | 1.47, 1.56 | 0.83, 0.77 |  | 1.53 |  | 54.85 | 42.42 | 24.93, 23.32 |  | 26.88 |
| 11-Ser |  | 4.32 | 3.73 |  |  |  |  | 58.05 | 63.91 |  |  |  |
| 12-His | 7.83 | 4.36 | 3.09, 2.95 |  |  |  |  | 57.31 | 30.86 |  |  |  |

**Supplementary Table S3: ^1^H and ^13^C chemical shifts of Pepcan-12 in NaP pH 3.0**

| Residue | NH | αH | βH | δH | εH | γH | zH | αC | βC | δC | εC | γC |
| --- | --- | --- | --- | --- | --- | --- | --- | --- | --- | --- | --- | --- |
| 1-Arg |  | 3.99 | 1.83 | 3.13 | 7.15 | 1.52 |  | 55.36 | 30.84 | 43.20 |  | 26.11 |
| 2-Val | 8.56 | 4.06 | 1.93 |  |  | 0.82, 0.84 |  | 62.15 | 32.76 |  |  | 20.45 |
| 3-Asp | 8.63 | 4.85 | 2.59, 2.79 |  |  |  |  | 51.36 | 38.59 |  |  |  |
| 4-Pro |  | 4.32 | 2.17, 1.84 | 3.75, 3.63 |  | 1.92 |  | 63.16 | 32.09 | 50.70 |  | 27.22 |
| 5-Val | 8.02 | 3.88 | 1.87 |  | 0.72 |  |  | 62.66 | 32.57 |  |  |  |
| 6-Asn | 8.24 | 4.60 | 2.71, 2.63 | 6.81, 7.52 |  |  |  | 52.73 | 38.67 |  |  |  |
| 7-Phe | 8.05 | 4.41 | 2.96, 3.04 | 7.15 | 7.27 |  | 7.22 | 58.43 | 39.39 |  |  |  |
| 8-Lys | 8.01 | 4.12 | 1.67, 1.61 | 1.56 | 2.89 | 1.25 | 7.44 | 56.47 | 32.88 | 29.02 | 42.05 | 24.74 |
| 9-Leu | 7.89 | 4.20 | 1.51 | 0.86, 0.79 |  | 1.52 |  | 55.09 | 42.21 | 24.77,23.52 |  | 26.87 |
| 10-Leu | 8.02 | 4.29 | 1.48, 1.55 | 0.83, 0.77 |  | 1.50 |  | 54.91 | 42.35 | 24.93, 23.28 |  | 26.93 |
| 11-Ser | 8.06 | 4.31 | 3.74 |  |  |  |  | 58.25 | 63.85 |  |  |  |
| 12-His | 8.15 | 4.51 | 3.07, 3.21 | 7.18 | 8.48 |  |  | 55.88 | 29.43 |  |  |  |

**Supplementary Table S4: ^1^H and ^13^C chemical shifts of Pepcan-12 in NaP 7.4**

| Residue | NH | αH | βH | δH | εH | γH | zH | αC | βC | δC | εC | γC |
| --- | --- | --- | --- | --- | --- | --- | --- | --- | --- | --- | --- | --- |
| 1-Arg |  | 3.80 | 1.74 | 3.11 |  | 1.49 |  | 55.76 | 31.93 | 43.28 |  | 26.36 |
| 2-Val |  | 4.11 | 1.93 |  |  | 0.82, 0.83 |  | 61.81 | 32.90 |  |  | 20.40,20.98 |
| 3-Asp | 8.47 | 4.79 | 2.40, 2.63 |  |  |  |  | 52.45 | 41.13 |  |  |  |
| 4-Pro |  | 4.29 | 2.19, 1.85 | 3.65, 3.78 |  | 1.93 |  | 63.23 | 32.13 | 50.69 |  | 27.23 |
| 5-Val | 8.11 | 3.87 | 1.91 |  | 0.74, 0.84 |  |  | 62.99 | 32.38 |  |  | 20.91,20.96 |
| 6-Asn | 8.19 | 4.59 | 2.70, 2.62 | 6.81, 7.56 |  |  |  | 52.97 | 38.76 |  |  |  |
| 7-Phe | 7.99 | 4.42 | 2.98, 3.04 | 7.14 | 7.26 |  | 7.23 | 58.31 | 39.25 |  |  |  |
| 8-Lys | 7.96 | 4.14 | 1.68, 1.61 | 1.57 | 2.88 | 1.26 |  | 56.32 | 32.95 | 29.03 | 42.07 | 24.72 |
| 9-Leu | 7.93 | 4.21 | 1.51 | 0.86, 0.80 |  | 1.52 |  | 55.09 | 42.23 | 24.77,23.55 |  | 26.89 |
| 10-Leu | 8.03 | 4.30 | 1.47, 1.56 | 0.83, 0.77 |  | 1.50 |  | 54.93 | 42.41 | 24.98, 23.28 |  | 26.97 |
| 11-Ser | 8.07 | 4.31 | 3.74 |  |  |  |  | 58.17 | 63.90 |  |  |  |
| 12-His | 7.84 | 4.36 | 3.10, 2.97 | 6.99 | 8.03 |  |  | 57.42 | 30.72 |  |  |  |

**Supplementary Table S5: ^1^H and ^13^C chemical shifts of Pepcan-9 in HFIP/NaP 50/50 % v/v pH 3.0**

| Residue | NH | αH | βH | δH | εH | γH | zH | αC | βC | δC | εC | γC |
| --- | --- | --- | --- | --- | --- | --- | --- | --- | --- | --- | --- | --- |
| 4-Pro |  | 4.58 | 2.23, 2.27 | 3.59 |  | 2.70 |  | 62.57 | 26.44 | 49.70 |  | 32.59 |
| 5-Val | 8.07 | 4.20 | 2.07 |  |  | 0.92, 1.06 |  | 63.03 | 33.41 |  |  | 20.40, 19.98 |
| 6-Asn | 7.80 | 4.89 | 2.88, 3.20 | 6.39, 7.22 |  |  |  | 52.59 | 38.06 |  |  |  |
| 7-Phe | 7.95 | 4.53 | 3.39, 3.21 | 7.41 |  |  |  | 60.57 | 39.21 | 51.18 |  |  |
| 8-Lys | 8.16 | 4.29 | 1.67, 2.09 | 1.92 | 3.20 | 1.64, 1.69 |  | 58.91 | 31.99 | 29.02 | 42.31 | 25.06 |
| 9-Leu | 7.63 | 4.40 | 1.87 | 1.08, 1.14 |  | 1.81 |  | 57.12 | 42.28 | 22.31,24.03 |  | 27.32 |
| 10-Leu | 7.70 | 4.45 | 1.94 | 1.08, 1.10 |  | 1.87 |  | 56.57 | 42.50 | 22.72,24.45 |  | 27.15 |
| 11-Ser | 7.87 | 4.55 | 4.03, 4.16 |  |  |  |  | 58.91 | 64.14 |  |  |  |
| 12-His | 8.08 | 4.81 | 3.48, 3.59 | 7.50 |  |  |  | 55.98 | 28.75 | 51.62 |  |  |

**Supplementary Table S6:^1^H and ^13^C chemical shifts of Pepcan-9 in HFIP/NaP 50/50 % v/v pH 7.4**

| Residue | NH | αH | βH | δH | εH | γH | zH | αC | βC | δC | εC | γC |
| --- | --- | --- | --- | --- | --- | --- | --- | --- | --- | --- | --- | --- |
| 4-Pro |  | 4.47 | 2.16, 2.21 | 3.07, 3.51 |  | 2.64 |  | 62.53 | 26.65 | 49.59 |  | 32.71 |
| 5-Val |  | 4.16 | 2.07 |  |  | 0.92, 1.04 |  | 63.09 | 33.22 |  |  | 20.37, 19.98 |
| 6-Asn | 7.88 | 4.83 | 3.11, 2.84 | 6.37, 7.17 |  |  |  | 52.91 | 38.05 |  |  |  |
| 7-Phe | 7.87 | 4.54 | 3.19, 3.38 | 7.52 | 7.39 |  |  | 60.15 | 39.11 | 69.67 | 69.59 |  |
| 8-Lys | 8.10 | 4.32 | 2.05, 1.79 | 1.64, 1.90 | 3.17 | 1.64 |  | 58.33 | 32.06 | 28.93 | 42.31 | 24.90 |
| 9-Leu | 7.63 | 4.38 | 1.79 | 1.05, 1.12 |  | 1.99 |  | 56.84 | 42.28 | 22.42,24.20 |  | 27.32 |
| 10-Leu | 7.58 | 4.49 | 1.93 | 1.05, 1.09 |  | 1.87 |  | 56.06 | 42.38 | 22.22,24.50 |  | 24.50 |
| 11-Ser | 7.83 | 4.55 | 4.02, 4.12 |  |  |  |  | 58.68 | 64.20 |  |  |  |
| 12-His | 7.94 | 4.64 | 3.49, 3.59 | 7.50 | 7.50 |  |  | 57.67 | 29.70 |  |  |  |

**Supplementary Table S7: ^1^H and ^13^C chemical shifts of Pepcan-12 HFIP/NaP 50/50 % v/v pH 3.0**

| Residue | NH | αH | βH | δH | εH | γH | zH | αC | βC | δC | εC | γC |
| --- | --- | --- | --- | --- | --- | --- | --- | --- | --- | --- | --- | --- |
| 1-Arg |  | 4.30 | 2.18 | 3.44 | 7.43 | 1.88, 1.91 |  | 55.76 | 31.15 | 43.27 |  | 26.31 |
| 2-Val | 8.42 | 4.36 | 2.25 |  |  | 1.13, 1.17 |  | 62.88 | 33.05 |  |  | 20.61,20.31 |
| 3-Asp | 8.49 | 5.25 | 3.03, 3.15 |  |  |  |  | 51.19 | 39.16 |  |  |  |
| 4-Pro |  | 4.54 | 2.52 | 4.02 |  | 2.20, 2.27 |  | 64.90 | 31.96 | 50.84 |  | 27.07 |
| 5-Val | 7.61 | 3.99 | 2.21 |  |  | 1.05, 1.15 |  | 64.94 | 32.05 |  |  | 20.43,20.31 |
| 6-Asn | 7.90 | 4.72 | 2.95, 3.00 | 6.73, 7.23 |  |  |  | 54.94 | 38.23 |  |  |  |
| 7-Phe | 8.05 | 4.52 | 3.30, 3.36 | 7.37 |  |  | 7.26 | 60.86 | 39.05 |  |  |  |
| 8-Lys | 8.21 | 4.23 | 2.17 | 1.92 | 3.19 | 1.71 |  | 58.91 | 31.93 | 28.90 | 42.31 | 24.82 |
| 9-Leu | 7.95 | 4.37 | 1.82, 1.99 | 1.06, 1.16 |  |  |  | 57.64 | 42.22 | 22.37,24.17 |  |  |
| 10-Leu | 7.94 | 4.42 | 1.80, 1.95 | 1.07, 1.09 |  |  |  | 56.77 | 42.45 | 22.19, 24.38 |  |  |
| 11-Ser | 7.87 | 4.48 | 3.89, 4.10 |  |  |  |  | 58.99 | 64.03 |  |  |  |
| 12-His | 8.02 | 4.77 | 3.48, 3.58 | 7.41 | 7.47 |  |  | 56.01 | 28.61 |  | 51.32 |  |

**Supplementary Table S8: ^1^H and ^13^C chemical shifts of Pepcan-12 HFIP/NaP 50/50 % v/v pH 7.4**

| Residue | NH | αH | βH | δH | εH | γH | zH | αC | βC | δC | εC | γC |
| --- | --- | --- | --- | --- | --- | --- | --- | --- | --- | --- | --- | --- |
| 1-Arg |  | 3.65 | 1.69, 1.74 | 3.19 | 7.19 | 1.52, 1.91 |  | 55.99 | 32.42 | 43.05 |  | 26.26 |
| 2-Val |  | 4.08 | 1.90 |  |  | 0.81, 0.84 |  | 62.19 | 32.75 |  |  | 20.33,19.80 |
| 3-Asp | 8.07 | 4.84 | 2.60, 2.67 |  |  |  |  | 52.11 | 41.41 |  |  |  |
| 4-Pro |  | 4.18 | 2.23 | 3.70 |  | 1.86, 2.27 |  | 60.61 | 31.70 | 50.54 |  | 26.86 |
| 5-Val | 7.68 | 3.69 | 1.99 |  |  | 0.78, 0.85 |  | 65.18 | 31.74 |  |  | 20.06,20.92 |
| 6-Asn | 7.94 | 4.36 | 2.65, 2.67 | 6.41, 7.08 |  |  |  | 54.80 | 37.99 |  |  |  |
| 7-Phe | 7.94 | 4.18 | 3.03, 3.05 | 7.06 |  |  | 7.26 | 64.84 | 38.75 | 50.85 |  |  |
| 8-Lys | 7.78 | 3.95 | 1.88 | 1.63 | 2.89 | 1.42 |  | 58.32 | 31.69 | 28.51 | 42.04 | 24.40 |
| 9-Leu | 7.72 | 4.06 | 1.49, 1.72 | 0.76 |  | 1.63 |  | 57.23 | 41.92 | 21.91 |  | 26.81 |
| 10-Leu | 7.59 | 4.16 | 1.47, 1.63 | 0.76, 0.77 |  |  |  | 56.04 | 42.26 | 23.93, 24.22 |  |  |
| 11-Ser | 7.51 | 4.17 | 3.57, 3.74 |  |  |  |  | 58.65 | 63.74 |  |  |  |
| 12-His | 7.54 | 4.30 | 3.09, 3.19 | 7.41 | 7.10 |  |  | 57.44 | 29.21 | 50.88 | 49.59 |  |


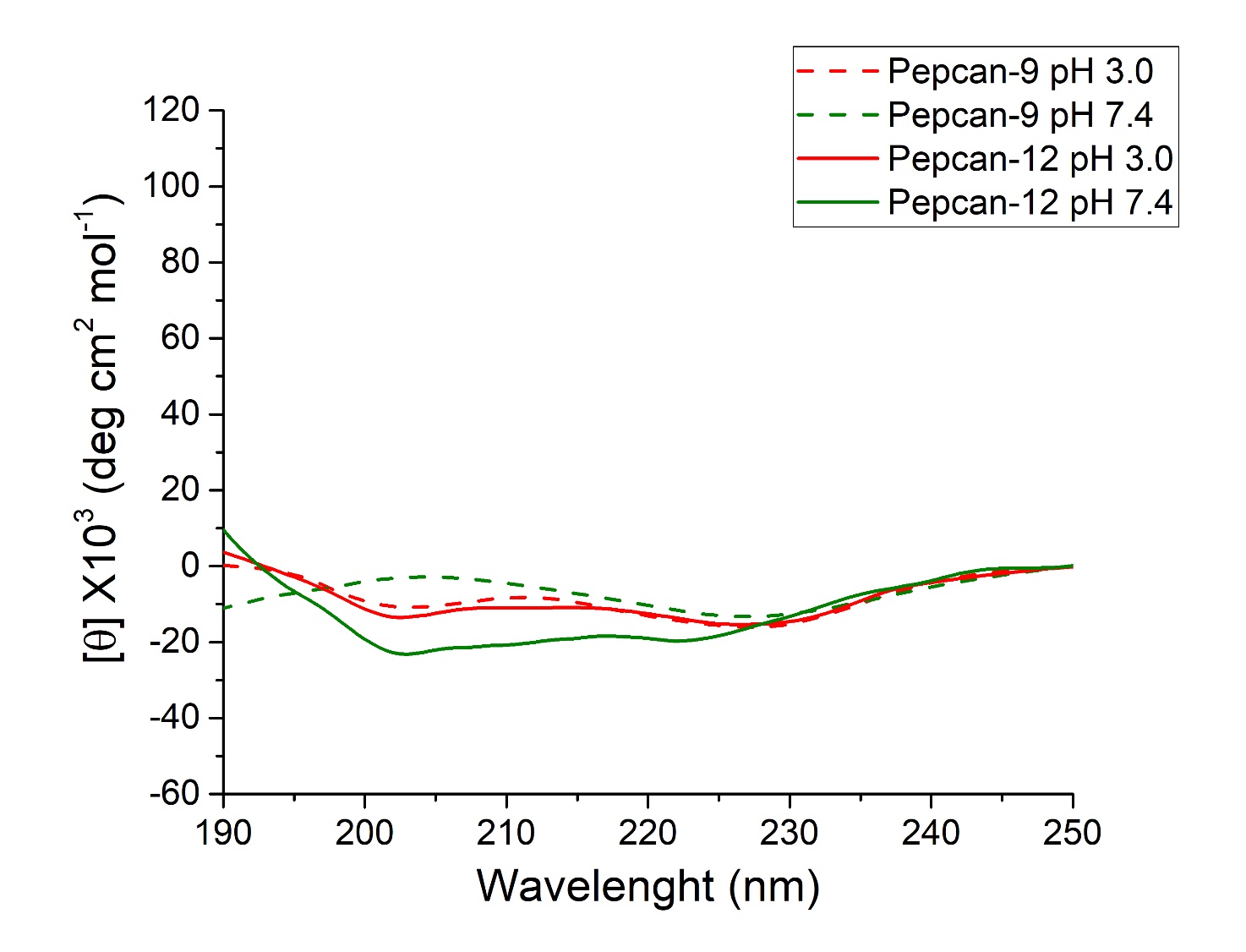


**Supplementary Figure S1:** CD spectra of Pepcan-9 (dashed lines) and Pepcan-12 (solid lines) obtained in NaP at pH 3.0 and 7.4.


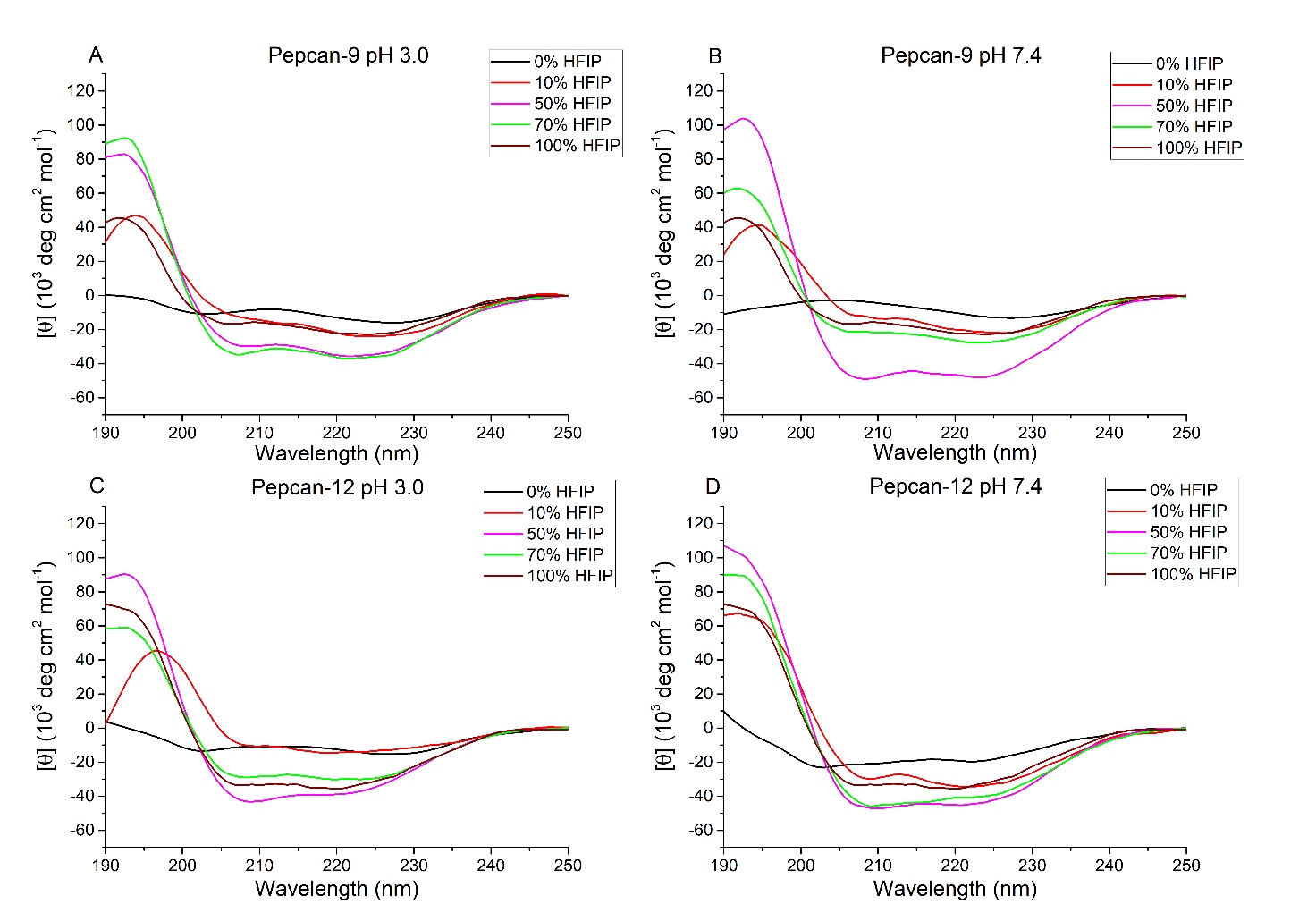


**Supplementary Figure S2:** Selection of CD spectra obtained for Pepcan-9 (panel A and B) and Pepcan-12 (panel C and D) in different NaP/HFIP mixtures at pH 3.0 and 7.4.


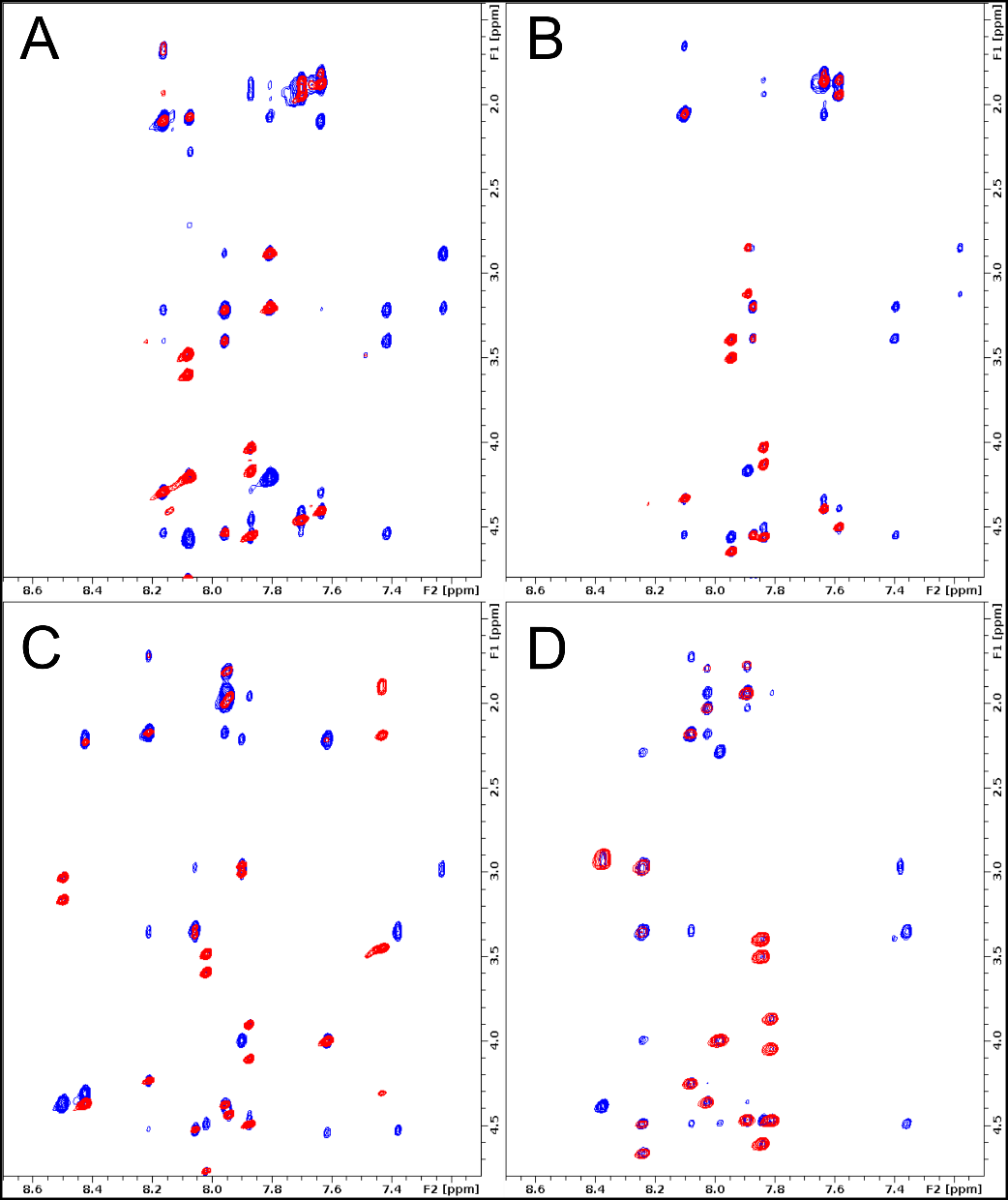


**Supplementary Figure S3:** Superimposition of TOCSY (red) and NOESY (blue) spectra obtained for Pepcan-9 in NaP/HFIP 50/50 v/v at pH 3.0 (panel A) and pH 7.4 (panel B), and for Pepcan-12 in NaP/HFIP 50/50 v/v at pH 3.0 (panel C) and pH 7.4 (panel D).


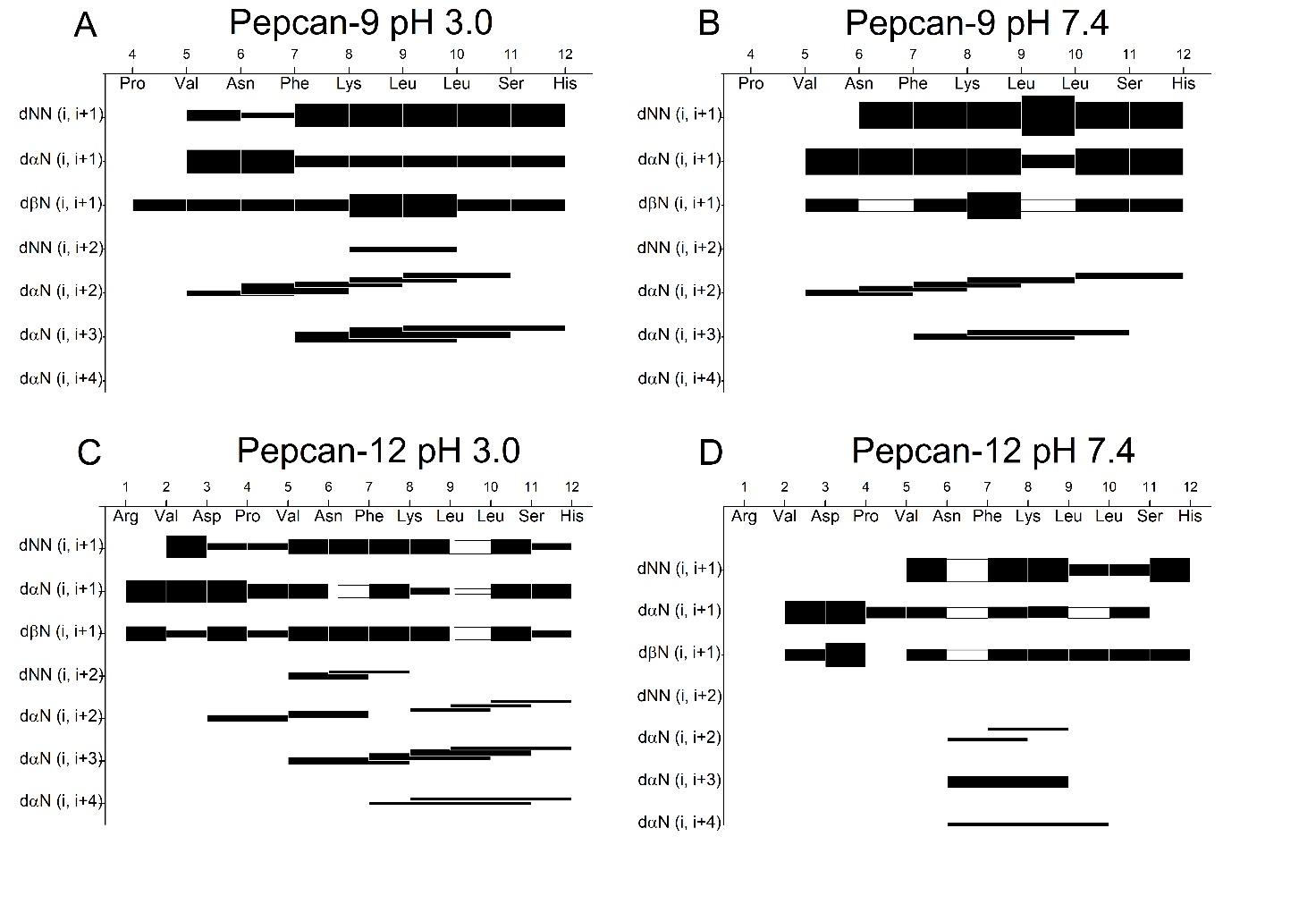


**Supplementary Figure S4:** Summary of sequential and medium-range NOEs obtained for Pepcan-9 in NaP/HFIP 50/50 v/v at pH 3.0 (panel A) and pH 7.4 (panel B), and for Pepcan-12 in NaP/HFIP 50/50 v/v at pH 3.0 (panel C) and pH 7.4 (panel D).


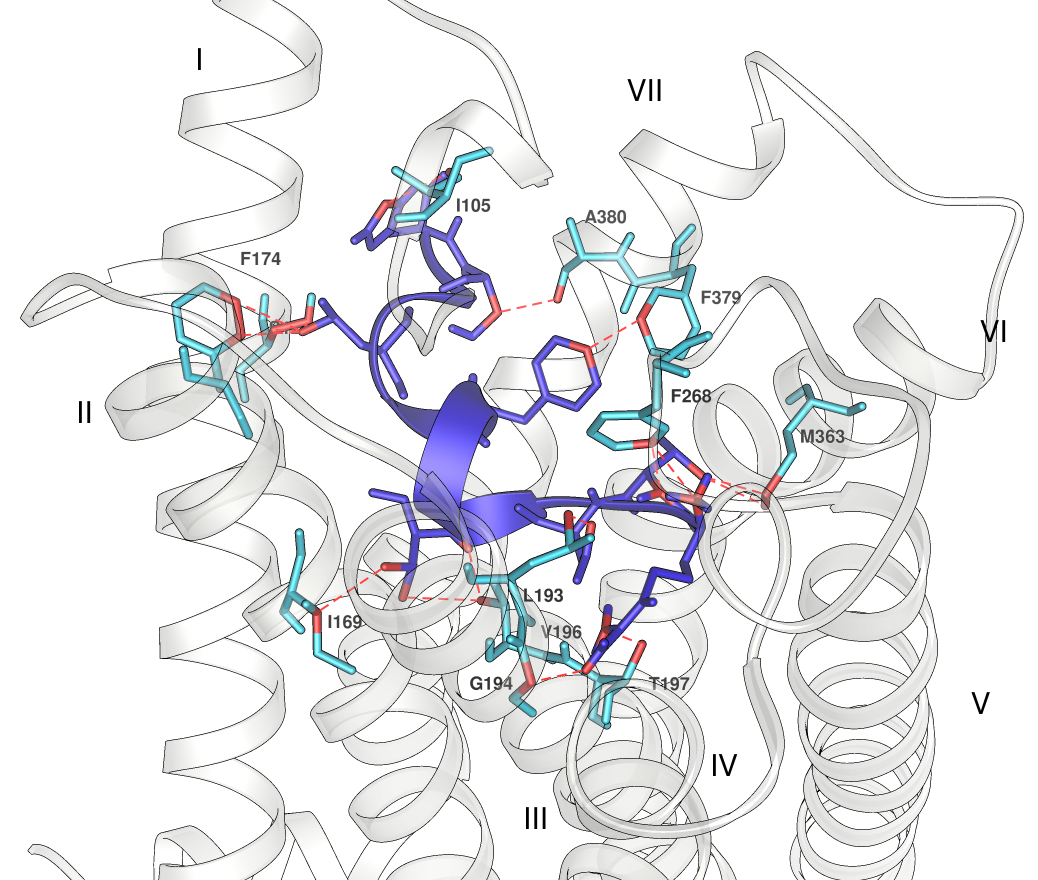
**Supplementary Figure S5:** Lowest energy docked pose of Pepcan-12 in the orthosteric binding site of CB1 (predicted binding energy 6.42 kcal/mol). Atom clashes (VdW radii overlap > 0.6 Å) between the ligand and the receptor are highlighted in red.
